# Fellowship training in microvascular surgery and post-fellowship practice patterns: a cross sectional survey of microvascular surgeons from facial plastic and reconstructive surgery programs

**DOI:** 10.1186/s40463-019-0342-y

**Published:** 2019-05-09

**Authors:** Douglas M. Bennion, Peter T. Dziegielewski, Brian J. Boyce, Yadro Ducic, Raja Sawhney

**Affiliations:** 1 Department of Otolaryngology, University of Iowa Hospitals and Clinics, Iowa City, IA USA; 2 Department of Otolaryngology, College of Medicine, University of Florida, 1600 SW Archer Drive, PO BOX 100264, Gainesville, FL 32610 USA; 3 Otolaryngology and Facial Plastic Surgery Associates, Fort Worth, TX USA

**Keywords:** Otolaryngology, Free tissue flaps, Microvascular surgery, Microsurgery, Graduate medical education, Facial plastic and reconstructive surgery fellowship, Head and neck oncology

## Abstract

**Background:**

There is a lack of published literature on the training in microvascular reconstructive techniques in facial plastic and reconstructive surgery (FPRS) fellowships or of the extent these techniques are continued in practice. This cross-sectional web-based survey study was conducted to describe the volume, variety, and intended extent of practice of free tissue transfers during fellowship and the post-fellowship pattern of microsurgical practice among FPRS surgeons in various private and academic practice settings across the United States.

**Methods:**

This survey was sent to recent graduates (*n* = 94) of a subset of U.S. Facial Plastic and Reconstructive Surgery fellowship programs that provide significant training in microvascular surgery.

**Results:**

Among survey respondents (*n* = 21, 22% response rate), two-thirds completed 20–100 microvascular cases during fellowship using mainly radial forearm, fibula, anterior lateral thigh, latissimus and rectus free tissue transfers. In post-fellowship practice, those who continue practicing microvascular reconstruction (86%) complete an average of 33 cases annually. The choice of donor tissues for reconstruction mirrored their training. They are assisted primarily by residents (73%) and/or fellows (43%), while some worked with a micro-trained partner, surgical assistant, or performed solo procedures. Interestingly, among those who began in private practice (29%), only half remained with that practice, while those who joined academic practices (71%) largely remained at their initial post-fellowship location (87%).

**Conclusions:**

These results provide the first formal description of the training and practice patterns of FPRS-trained microvascular surgeons. They describe a diverse fellowship training experience that often results in robust microvascular practice. The maintenance of substantial microsurgical caseloads after fellowship runs counter to the perception of high levels of burnout from free tissue transfers among microvascular surgeons.

**Trial Registration:**

This study was approved as exempt by the University of Florida Institutional Review Board (#201601526).

## Introduction

Within the field of Otolaryngology – Head and Neck Surgery, several training pathways have developed through which surgeons are trained in microvascular head and neck reconstructive surgery, including free tissue transfer (FTT). Commonly included as part of Head & Neck Oncology fellowships, training in microvascular surgery techniques in certain Facial Plastic and Reconstructive Surgery (FPRS) fellowships has also become well-established over the last several decades [1, 2]. Formal descriptions of such training during FPRS fellowships are lacking, leaving an unclear understanding of which programs offer such training and to what extent. Microvascular surgery is an area of training to which residents have widely variable exposure prior to fellowship [3], making more difficult the assessment of readiness for microvascular fellowships. After FPRS fellowship, practicing microvascular and reconstructive surgeons pursue a wide variety of surgical techniques, caseloads, and practice models, and no previously published literature exists to describe their patterns of practice, including the continuance of microvascular and reconstructive surgery, which is suggested to carry higher risk of physician burnout [4]. Given the lack of formal assessments of this information previously, the aims of this descriptive study were to assess the breadth of fellowship training and to describe post-fellowship practice patterns of FPRS-trained microvascular surgeons.

## Participants and study design

This cross-sectional web-based survey study was conducted among recent graduates of a subset of U.S. Facial Plastic and Reconstructive Surgery fellowship programs that provide significant training in microvascular surgery. Through a process of email and telephone inquiry, nine responding programs were identified as providing training in microvascular surgical techniques. The survey population was composed of recently graduated fellows from these programs.

The survey instrument (see Online-Only Supplement) was designed to include subsets of questions about fellowship training and about post-fellowship practice. The list of potential participants and email addresses were generated using a combination of direct inquiry to program coordinators, information available on program websites, and use of publicly available contact information. Invitations to voluntarily participate were sent electronically using a Qualtrics web-based survey tool to 94 surgeons for whom email addresses were available and who had completed fellowship training after 1996. An additional 23 graduates did not have email addresses that were made available. Follow-up survey email invitations were sent at 4 weeks and 6 weeks with a total of 21 responses (22% response rate). It was not possible to confirm receipt of the survey among non-responders given the substantial potential for outdated addresses or unattended email inboxes. Data from all respondents were included and there were no partial completions. Descriptive statistical analyses were performed using Microsoft Excel.

## Results

### FPRS fellowship training in microvascular surgical techniques

**Fig. 1:**
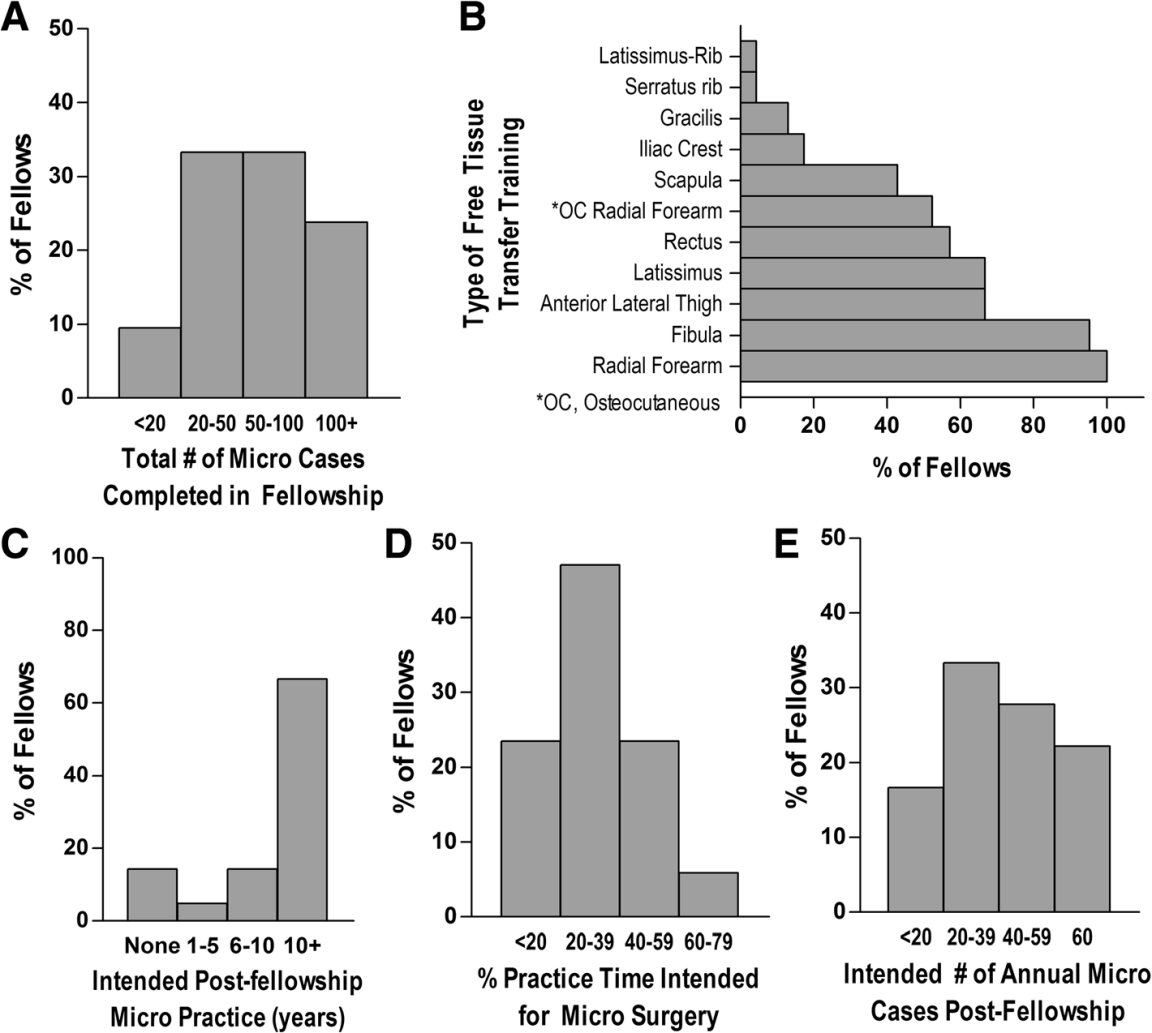
Summary of training in microvascular head and neck reconstructive surgical techniques during facial plastic and reconstructive surgery fellowship. The total number (**a**) and variety (**b**) of microvascular cases completed during fellowship. The respondents’ intended number of years (**c**), percentage of practice time (**d**), and number of annual cases (**e**) to perform microvascular surgery upon completion of fellowship

We received 21 voluntary responses from FPRS-trained microvascular surgeons who completed fellowships between 1996 and 2014 (median year, 2011). In response to questions about their experience during fellowship, two-thirds of survey respondents reported completing 20–100 microvascular cases during fellowship, with 1 in 4 completing more than 100 cases (Fig. 1a). In performing FTT procedures, every respondent was trained to perform radial forearm free flaps (100%), and the large majority were also trained in fibula (95%), anterior lateral thigh (67%), latissimus (67%), and rectus FTTs (57%, Fig. 1b). When asked about their intentions for post-fellowship performance of microvascular surgical cases, 14% did not plan to do so, citing lifestyle concerns, lack of interest, and plans to join partners who already performed FTTs, while 2 of 3 planned to pursue microvascular surgery for 10+ years (Fig. 1c). They intended to devote, on average, a little less than half of their time performing an average of 38 cases per year, though this varied widely (Fig. 1d&e).

### Post-fellowship practice patterns

**Fig. 2:**
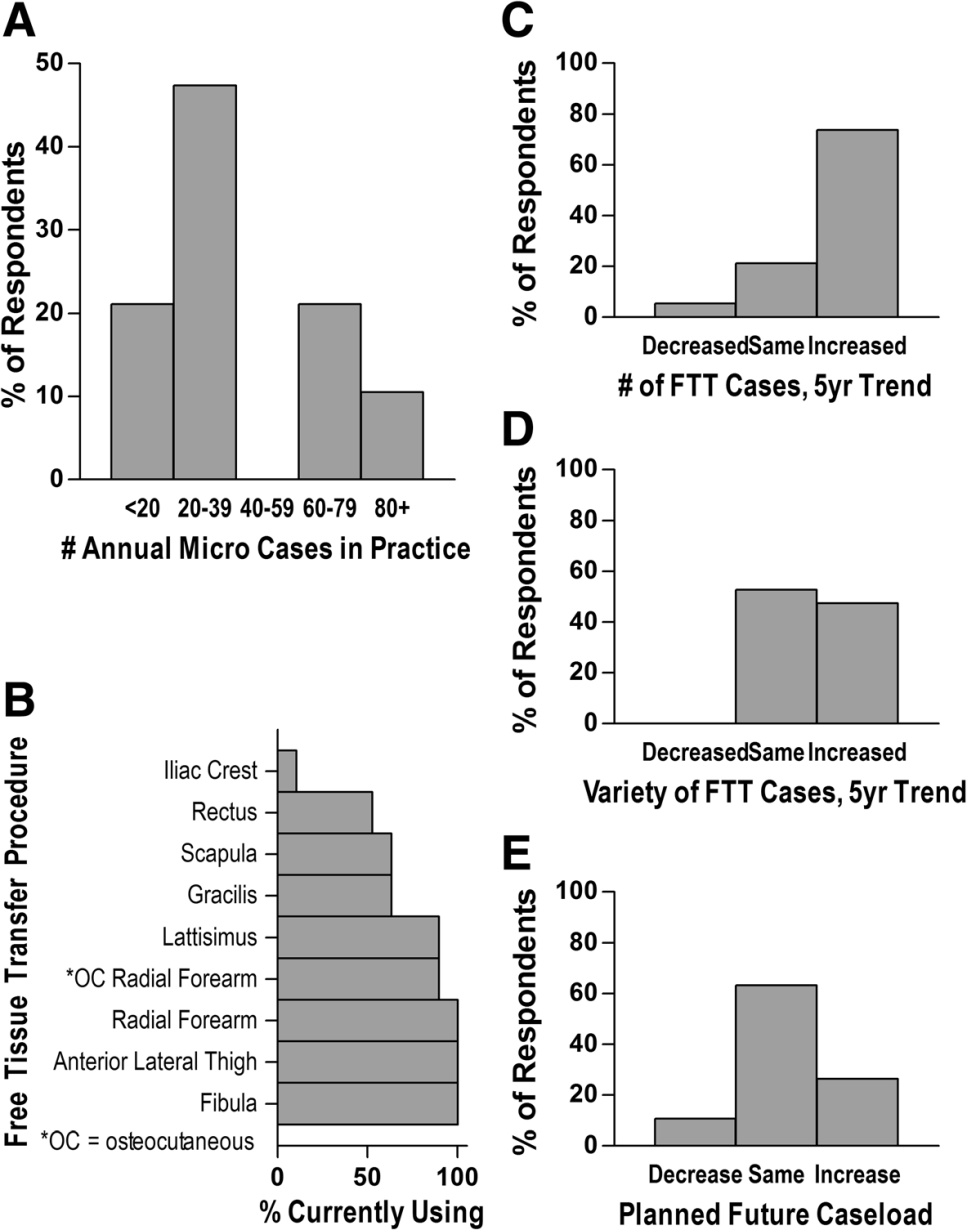
Summary of post-fellowship microvascular surgery practice patterns. The number (**a**) and variety (**b**) of microvascular cases completed annually in practice. The previous five year trend in number (**c**) and variety (**d**) of microvascular cases performed, and the anticipated change in future caseload (**e**) as a percentage of respondents

In post-fellowship practice, those who continued practicing microvascular reconstruction (86%) reported completing an average of 33 cases annually (Fig. 2a). Interestingly, surgeons in practice diverged into two groups: those performing less than 40 cases per year (68%) and those performing more than 60 cases per year (32%), with no respondents completing between 40 and 60 per year. The choice of donor tissues for reconstruction mirrored their training (e.g. radial forearm, fibula, anterior lateral thigh, Fig. 2b). In looking back on the previous five years, three out of four respondents had experienced an increase in the number of FTTs they performed, with half also reporting an increased variety in their free flap cases (Fig. 2c&d). Nearly two-thirds planned to maintain their current caseload, with one in four reporting plans to increase caseloads over the next five years (Fig. 2e). The other 10% planned to decrease the number of microvascular cases in favor of increasing cases involving Moh’s reconstruction, facial paralysis, and cosmetics.

**Fig. 3:**
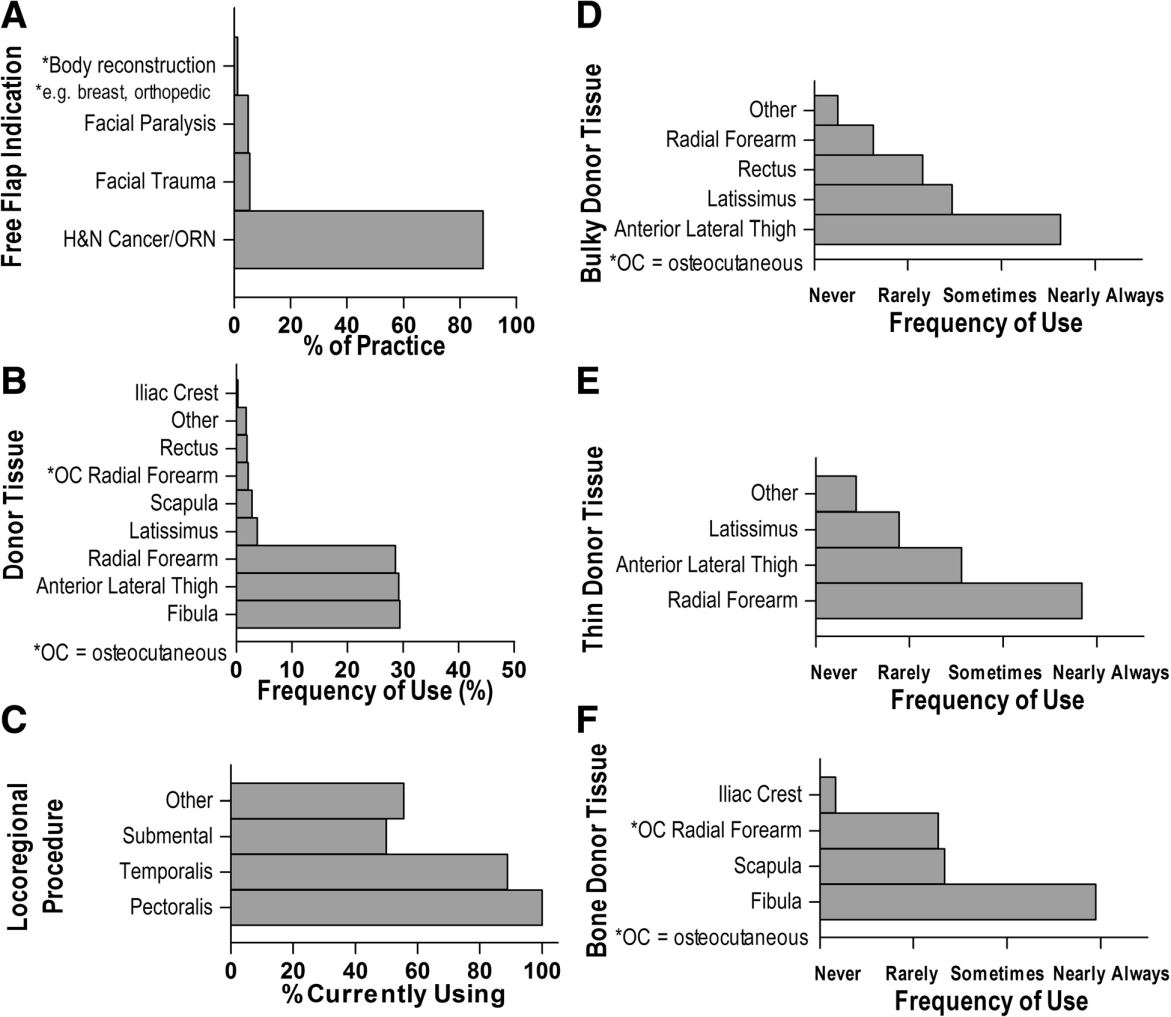
Description of free tissue transfer indications and techniques in practice. The indication for performing free flap procedures as a percentage of practice (**a**). The type of donor tissue as an overall percentage of frequency used (**b**) during free flap procedures. For locoregional cases, the percentage of respondents using each flap (**c**). Specific frequency of tissue use when needed for reconstructing bone **d**), bulky tissue (**e**), or thin tissue (**f**)

FTTs in practice were mainly indicated for the treatment of head and neck cancers and osteoradionecrosis (88% of cases, Fig. 3a). When choosing donor tissue for transfer, surgeons reported choosing at nearly equal frequencies from fibula (29.5%), anterior lateral thigh (29.2%), and radial forearm (28.6%, Fig. 3b). In performing locoregional construction, they turned to pectoralis, temporalis, and submental flaps, among others (Fig. 3c). The most commonly selected donor tissue when there was a need for free flap reconstruction of bone was fibula, for bulky tissue was anterior lateral thigh, and for thin tissue was radial forearm (Fig. 3d-f).

**Fig. 4:**
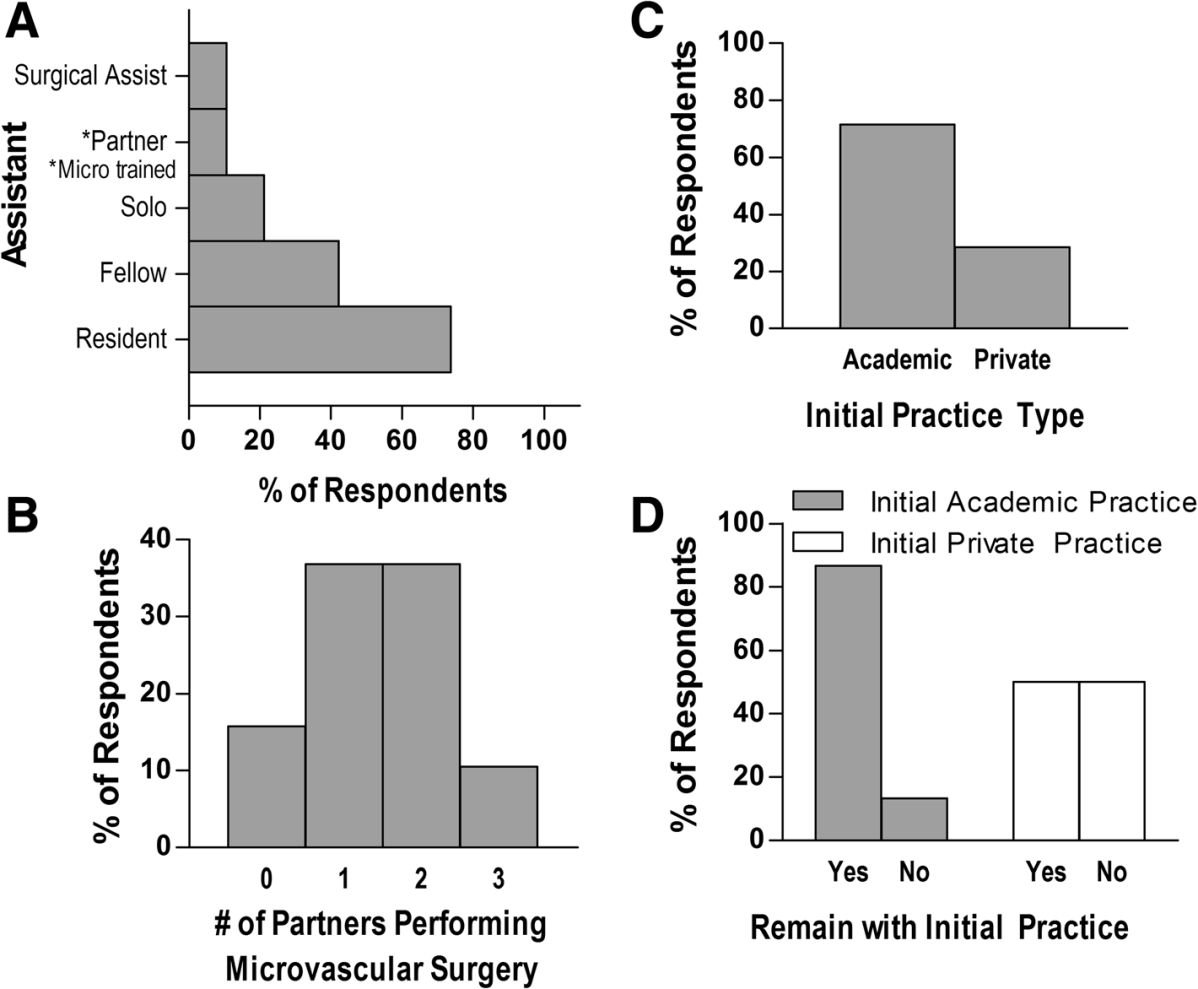
Description of practice settings. The kind of surgical assistant used (**a**) and number of partners performing microvascular surgery (**b**) as a percentage of respondents. The percentage of respondents who joined an academic versus private practice out of fellowship (**c**) and whether they remained with their initial practice (**d**) after joining an academic (grey bars) or private (white bars) practice

In performing these procedures, respondents were most often assisted by a resident (73%) and/or fellow (43%), while some worked with a micro-trained partner, surgical assistant, or performed solo procedures (Fig. 4a). Greater than two-thirds of respondents reported having either one or two partners who also perform microvascular surgery (Fig. 4b). Interestingly, among those who began in private practice (29%) out of fellowship, half had experienced a subsequent move to a different practice, while those who joined academic practices (71%) largely remained at their initial post-fellowship location (87%, Fig. 4c&d).

## Discussion

### Summary of main results

These results of this descriptive survey study detail the training and practice patterns of a subset of facial plastics-trained microvascular surgeons. Respondents reported the attainment of broad training in the use of vascularized flaps, with focus a on radial forearm, fibula, and anterior lateral thigh, techniques that they continued to apply regularly in practice. Importantly, microvascular surgical practice is well-maintained among the subset of facial plastic and reconstructive surgeons surveyed, with 86% continuing to perform FTTs beyond fellowship. Most of these surgeons (71%) join and stay in academic practice, working with multiple partners, residents, fellows, and other assistants.

### Study limitations and potential biases

This cross-sectional study has several important limitations. The number of responses provides limited power to identify correlations between aspects of an individual’s fellowship training and their future practice patterns, though the overall the conclusion that microvascular training (Fig. 1a&b) translates consistently into microvascular practice (Fig. 2a&b) is well-supported. The inability to accurately identify the receipt status of the electronic invitation to participate among members of the study population (see Methods) complicates the response rate calculation, and likely means that the response rate was higher than reported. Further, practice demographics from our sample compare favorably with those from a larger published study among microvascular and reconstructive free-flap head and neck surgeons, demonstrating very similar numbers of free-flap procedures performed annually, years in practice, and proportion in academic versus private practice [5]. However, these considerations do not eliminate the potential for non-response bias in our study. We also cannot account for the potential of recall bias given respondents’ self-reporting about activities that were, in some cases, years prior. It is also important to note that the relatively recent completion of fellowship training (median year of 2011) among respondents provides a limited picture of the longitudinal career paths for microvascular facial plastic surgeons, and future study of this group should seek to incorporate responses from more tenured practitioners.

### Implications for clinical practice and research

Certain notable aspects of microvascular surgery in practice may contribute to physician burnout, as reported in a 2010 cross-sectional study of burnout among microvascular surgeons [5]. Respondents in that study, who were largely derived from academic practices, identified having too little time to do research, too little administrative time, and low levels of control over professional life as leading stressors. Among our cohort, who also largely joined academic practices where they tended to remain (Fig. 4c&d), respondents who indicated that they planned to eliminate or decrease microvascular cases from their practice gave the following reasons: “too labor intensive to do for a long period of time; planning to branch out to other fields of plastic surgery to lessen the burden physically/mentally/emotionally; free flap cases can contribute to burn out when done at my current volume of around 60 free flaps/yr.” Another wrote that they were disinclined due to “lifestyle concerns, sick patient population, urgent add on cases requiring rearrangement of schedule, cases extending into the evening/night, being on call all the time as the sole microvascular surgeon.” Importantly, 86% of our respondents continue to carry substantial microvascular caseloads, with 90% planning to maintain or increase this load over the next five years (Fig. 2e). This is in keeping with results from a 2007 study that found that 71.6% of U.S. academic microvascular surgeons continued to performed free-flap procedures [6]. Overall, these findings are in support of the suggestion that the perception of high levels of burnout among microvascular free flap surgeons in general may be overstated [5], perhaps especially among those who are FPRS-trained. The toll over the long run is not fully captured in our study population, however. Additional study and longer-term follow-up is needed to characterize what effect these concerns may have on the longevity of these surgeons in performing FTTs.

The results from this descriptive study have the potential to inform the decisions of trainees considering subspecialty training at FPRS fellowship programs that perform microvascular surgery, especially in light of their variable levels of microvascular exposure during residency [3]. They may also be of interest to those overseeing their fellowship training in guiding decisions about the volume and variety of procedures fellows may expect. For those currently in practice, this report provides a benchmark for comparison in the evolving field of otolaryngologic reconstructive microvascular surgery.

## Abbreviations

FPRS: Facial plastic and reconstructive surgery
FTT: Free tissue transfer
